# Oral health conditions and frailty in Mexican community-dwelling elderly: a cross sectional analysis

**DOI:** 10.1186/1471-2458-12-773

**Published:** 2012-09-12

**Authors:** Roberto Carlos Castrejón-Pérez, S Aída Borges-Yáñez, Luis M Gutiérrez-Robledo, J Alberto Ávila-Funes

**Affiliations:** 1 Programa de Maestría y Doctorado en Ciencias Médicas, Odontológicas y de la Salud, Universidad Nacional Autónoma de México, Ciudad Universitaria, Av. Universidad 3000, Del. Coyoacán, C.P. 04510, Distrito Federal, México; 2 Coordinación de Salud Pública Bucal, División de Estudios de Posgrado e Investigación, Facultad de Odontología, Universidad Nacional Autónoma de México, Ciudad Universitaria, Av. Universidad 3000, Del. Coyoacán, C.P. 04510, Distrito Federal, México; 3 Instituto Nacional de Geriatría, Blvd. Adolfo Ruíz Cortines 2767, Col. San Jerónimo Lídice, Del. Magdalena Contreras, C.P. 10200, Distrito Federal, México; 4 Clínica de Geriatría, Instituto Nacional de Ciencias Médicas y Nutrición,“Salvador Zubirán”, Vasco de Quiroga 15, Col. Sección XVI, Del. Tlalpan, C.P. 14000, Distrito Federal, México

**Keywords:** Elderly, Oral health, Frailty syndrome, Utilization of dental services

## Abstract

**Background:**

Oral health is an important component of general well-being for the elderly. Oral health-related problems include loss of teeth, nonfunctional removable dental prostheses, lesions of the oral mucosa, periodontitis, and root caries. They affect food selection, speaking ability, mastication, social relations, and quality of life. Frailty is a geriatric syndrome that confers vulnerability to negative health-related outcomes. The association between oral health and frailty has not been explored thoroughly. This study sought to identify associations between the presence of some oral health conditions, and frailty status among Mexican community-dwelling elderly.

**Methods:**

Analysis of baseline data of the Mexican Study of Nutritional and Psychosocial Markers of Frailty, a cohort study carried out in a representative sample of people aged 70 and older residing in one district of Mexico City. Frailty was defined as the presence of three or more of the following five components: weight loss, exhaustion, slowness, weakness, and low physical activity. Oral health variables included self-perception of oral health compared with others of the same age; utilization of dental services during the last year, number of teeth, dental condition (edentate, partially edentate, or completely dentate), utilization and functionality of removable partial or complete dentures, severe periodontitis, self-reported chewing problems and xerostomia. Covariates included were gender, age, years of education, cognitive performance, smoking status, recent falls, hospitalization, number of drugs, and comorbidity. The association between frailty and dental variables was determined performing a multivariate logistic regression analysis. Final models were adjusted by socio-demographic and health factors

**Results:**

Of the 838 participants examined, 699 had the information needed to establish the criteria for diagnosis of frailty. Those who had a higher probability of being frail included women (OR = 1.9), those who reported myocardial infarction (OR = 3.8), urinary incontinence (OR = 2.7), those who rated their oral health worse than others (OR = 3.2), and those who did not use dental services (OR = 2.1). For each additional year of age and each additional drug consumed, the probability of being frail increased 10% and 30%, respectively.

**Conclusions:**

Utilization of dental services and self-perception of oral health were associated with a higher probability of being frail.

## Background

The mouth and its associated structures participate actively in protective and biological functions, and are essential for social activities [1-3]. The aging people experience a wide variety of oral health problems, such as loss of teeth, edentulism, periodontitis, coronal and root caries, oral mucosal lesions, utilization of nonfunctional dental prostheses (either partial or complete), xerostomia, and chewing problems, among others [4-8]. These problems begin earlier in life and can promote a decline in general health because reduced nutrient intake, pain, and low quality of life [4,9-16].

Dental problems in the elderly are associated with modifications in food selection, changes in nutrient intake [6,7,10,13,17,18], chronic conditions such as diabetes [19], and with cardiovascular problems [20-22]. Oral health problems can be considered chronic diseases because their prevalence and duration; besides, risk factors are common with those of other chronic diseases, such as diabetes or cardiovascular problems [2,6,13,23-25].

Frailty syndrome is characterized by decreased resilience and physiological reserves, and is generally associated with an increased risk for disability, dependence, falls, hospitalization, and death [26-29]. Frailty results from excessive demands imposed on a system with a diminished capacity [30,31]; its prevalence ranges from 3–30% in people ≥65 years old [26,30,32,33].

**Figure 1:**
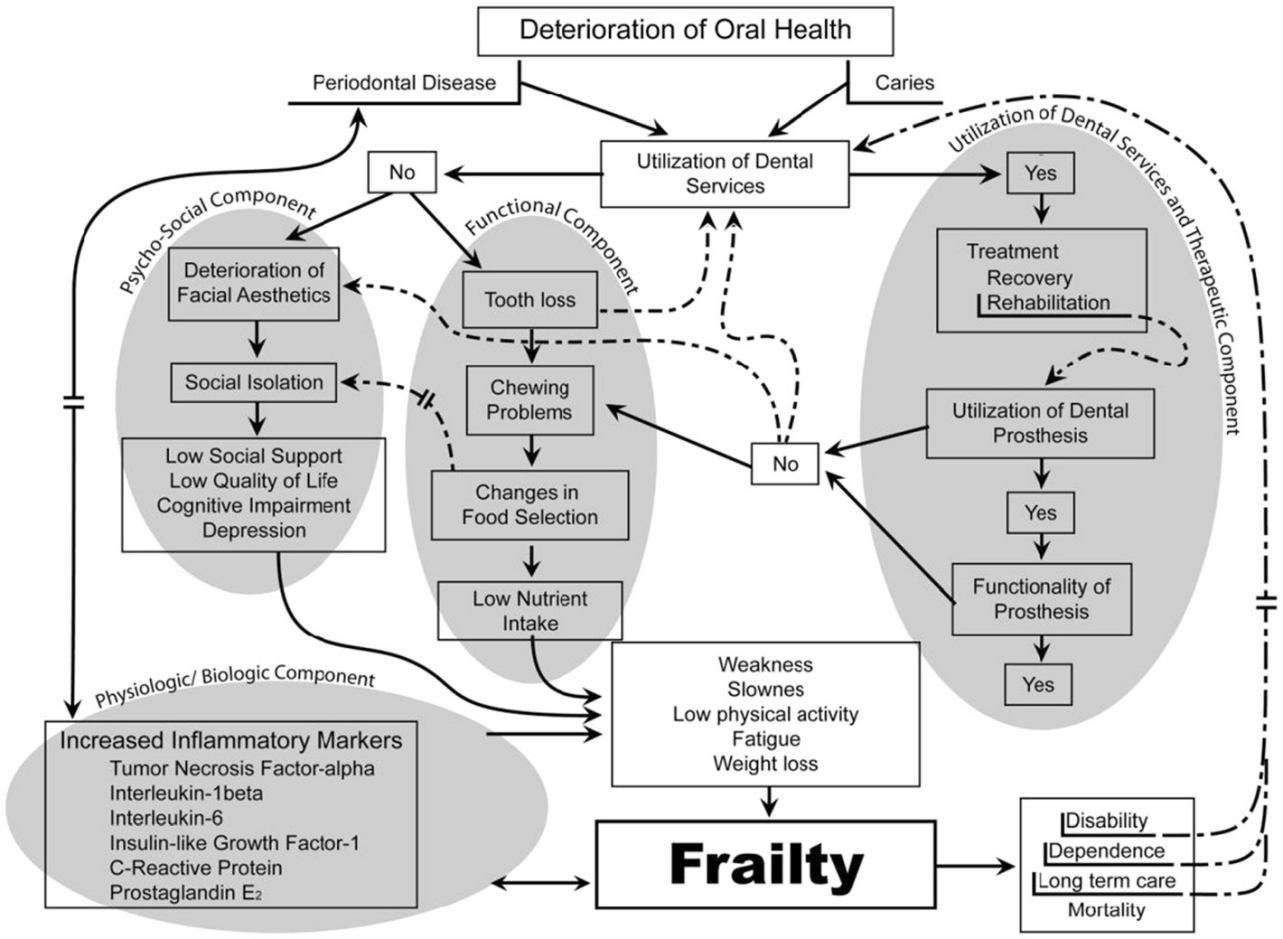
Proposed model for oral health and frailty.

To explain the approaches relating oral health status and the use of dental services with frailty; it is possible to build a four elements model. The first element is related to therapeutic decisions and the use of dental services, the second is focused on the functional ability of the mouth, the third one refers to the psychosocial process of the subject, and the fourth will focus on the cellular and physiological phenomena related with the inflammatory response. These components emerge from the deterioration of oral health due to either caries or periodontal disease, which can lead to the need for dental care [1-4], which may be used or not by the subjects leading to different outcomes affecting functionality and quality of life. Moreover, the first and fourth elements could also participate in both ways, periodontal inflammatory processes contribute to an increase in the levels of local and systemic inflammatory mediators and the increment of inflammation due to metabolic alterations contributes to the development or increased severity of periodontal disease, on the other hand, the reduction of mobility and development of disability or dependence consequence of frailty will affect the utilization of dental services because of problems of accessibility; this situation could contribute to the continuous deterioration of oral health (Figure 1). 

It has been reported that the use of complete dentures and reports of chewing problems and loss of teeth are associated with fatigue (a component of frailty) and mortality in the elderly [34,35]. However, associations between other oral health conditions and frailty have not been explored.

The aim of our baseline data analysis was to determine the association between frailty and the presence of oral health conditions such as edentulism, number of teeth present, utilization and/or functionality of removable partial dentures (RPD) or complete dentures (CD), severe periodontitis, xerostomia, self-reported chewing problems, self-perception of oral health, and utilization of dental services during the previous year. The results were adjusted by gender, age, years of education, smoking status, history of myocardial infarction, previous stroke, hypertension, diabetes, osteoporosis, arthritis, urinary incontinence, falls, hospitalization, and number of drugs.

We tested the hypothesis that poor oral health conditions are associated with an increased probability of frailty in community-dwelling elderly.

## Methods

The study sample is a subset of the participants of the Mexican Study of Nutritional and Psychosocial Markers of Frailty (the Coyoacan cohort), a prospective cohort study aiming to evaluate the nutritional and psychosocial determinants of frailty among Mexican community-dwelling elderly, conducted by the Department of Geriatrics of the National Institute of Medical Sciences and Nutrition “Salvador Zubirán” (INCMNSZ) of Mexico in collaboration with the National Institute of Public Health (INSP), the Department of Dental Public Health, the Graduate Studies and Research Division of the Dental School at the National Autonomous University of Mexico (UNAM), the Department of Health of the Government of the Federal District of Mexico, and the National Institute of Geriatrics of the Mexican Ministry of Health.

### Population

The study population consisted of 33 347 persons who lived independently in the District of Coyoacán, one of the 16 districts of Mexico City. They all received support from the Food Support, Medical Care, and Free Drugs Program, a local government program that serves 95% of elderly aged 70 and older. This district was selected because it was easily accessible and was home to citizens from a wide range of socioeconomic strata.

The sample was chosen by a random sampling procedure, stratified by age and gender, and we ensured a sample size that could reliably estimate a prevalence rate of frailty of at least 14% among participants with α = 5% and β = 20% (n = 1294).

Baseline data were collected during 2008 and 2009 in two stages. During the first stage, an interview was conducted using a standard questionnaire; during the second stage, a clinical (medical and dental) evaluation was carried out. The interview and clinical evaluation were carried out in the participant’s home. The study protocol was approved by the Ethical Committee of the INCMNSZ. Each participant signed an informed consent and was free to refuse a specific part of the examination.

### Variables

Frailty was defined according to the construct derived from the Cardiovascular Health Study [26] and validated in other studies [32,36]. Frail persons were identified as having three or more of the five following components: unintentional weight loss, poor endurance and energy, low physical activity, slowness, and weakness. These components were defined as follows:

➢ *Weight loss*: self-reported weight loss of 5 kg or more in the last 6 months was considered positive for this item.

➢ *Poor endurance and energy*: Self-report of exhaustion was assessed by two questions from the Center for Epidemiologic Studies-Depression scale (CES-D) [37]: “*I felt that everything I did was an effort*” and “*I could not get going*”. Participants were asked: *“How often, in the last week, did you feel this way?”* and the answer was quoted as follows: 0 = rarely or none of the time; 1 = some or a little of the time; 2 = a moderate amount of the time; or 3 = most of the time. Participants answering “2” (a moderate amount of the time) or “3” (most of the time) to either of these two questions were considered as frail for this criterion.

➢ *Low physical activity*: The Physical Activity Scale for the Elderly questionnaire (PASE) [38] was used to assess physical activity. Participants who scored in the lowest quintile adjusted for gender were categorized as frail for the low physical activity criterion.

➢ *Slowness*: A response “yes” or “can’t do” to any of the following questions was used to estimate slowness: *Because of a health problem,* “*do you have difficulty walking one block?*” or “*do you have difficulty with climbing several flights of stairs without resting?*”

➢ *Weakness*: participants who answered “yes” to the question *Because of a health problem,* “*do you have difficulty with lifting or carrying objects weighting over 5 kg, like a heavy bag of groceries?*” were categorized as frail for this criterion.

### Oral health variables

*Number of teeth.* (0–32 teeth): Number of natural teeth present in the mouth.

*Dental condition* (edentulism/partial edentulism/completely dentate): Edentulism is defined as the absence of all natural teeth; partial edentulism is defined as having one to 24 natural teeth; completely dentate is defined as having ≥25 teeth [39].

*Utilization of removable partial dentures (RPD) or complete dentures (CD)* (Yes/No): Determined during the clinical examination. Participants were asked to show their RPD or CD to the examiner; both those who were using dentures at the time of the evaluation and those who showed but did not use them were classified as denture users.

*Functionality of Removable Partial Dentures or Complete Dentures* (Functional/Nonfunctional): Evaluation of the stability, retention, occlusion, extension, and integrity of the dental prosthesis, as proposed by Ettinger [40]. Dentures failing in one or more of the above criteria were considered nonfunctional. Prostheses were also considered nonfunctional when the subjects expressed that they did not wear their removable prostheses regularly.

*Severe periodontitis* (Yes/No): Periodontitis status was evaluated using a modified version of the Periodontal Screening and Recording Index (PSR) [41]; we measured the clinical attachment loss of periodontal ligament by probing six sites per tooth (distobuccal, midbuccal, mesiobuccal, mesiolingual, midlingual, and distolingual) on all teeth present in the mouth, recording the highest PSR score on each tooth. We classified each participant according to the following criteria: Severe periodontitis was defined as having at least one tooth with a PSR score of 3 (3.5–5.5 mm of attachment loss) and furcation involvement or gingival recession of ≥3.5mm, or at least one tooth with a PSR score of 4 (>5.5 mm of attachment loss); the absence of severe periodontitis was defined by all teeth having PSR scores of 0 thru 3 (0–5.5 mm of attachment loss) without furcation involvement or gingival recession.

The following variables were assessed during the interview: self-perception of oral health compared with other persons of the same age (Better/Equal/Worse), utilization of dental services during the last year (Yes/No), xerostomia (Yes/No), and self-reported chewing problems (Yes/No).

### Covariates

The following socio-demographic and health variables were collected by interview: age, gender, education level (years), current and past smoking (Yes/No); cognitive impairment as evaluated by the Mini-Mental State Examination (MMSE) (≤18/19-30) [42]; urinary incontinence (Yes/No); falls three times or more in the previous 12 months (Yes/No); hospitalization in the previous 12 months (Yes/No), and the number of medications per day.

The presence or absence of six self-reported diseases was interrogated for their relationship with dental conditions and frailty: myocardial infarction, stroke, hypertension, diabetes, osteoporosis, and arthritis.

### Interview

**Figure 2:**
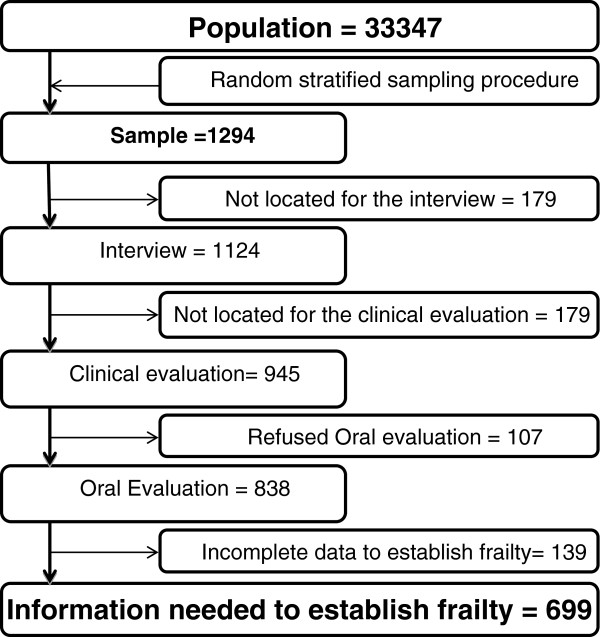
**Flowchart.** Flow of participants.

The interview was completed by 86.9% of the study sample (n = 1124); 24 persons could not be reached, 37 refused to participate, 18 were deceased, and the remaining 91 did not participate for other reasons. The interviews were performed by trained and standardized interviewers (Figure 2).

### Clinical evaluation

The participants were visited by an interdisciplinary team, consisting of a physician, a nutritionist, and a dentist. The subjects underwent a comprehensive geriatric health evaluation including examination of functional status, pharmacological treatments, physical performance, nutritional status and oral condition.

The oral evaluation included determination of the number of teeth present in the mouth, dental condition, use and functionality of RPD and CD, and presence of severe periodontitis. The dentists who carried out this evaluation were students of the dental school at UNAM; they were previously standardized in periodontal evaluation (Kappa = 0.7) and evaluation of the functionality of RPD and CD (Kappa = 0.9). The clinical evaluation was performed with artificial light, a dental mirror (#5), and a periodontal probe (CP11.5B, Hu-Friedy®), which had been previously sterilized; infection control procedures were strictly followed.

The overall clinical evaluation was completed by 945 subjects, and 107 subjects refused the oral evaluation; 74.5% (838/1124) of participants completed both the interview and the oral clinical evaluation (Figure 2).

### Analysis

Variables were described using frequencies and proportions or arithmetic means and standard deviations (SD) when appropriate. Univariate analyses testing oral health conditions (and covariates) with frailty status were carried out using chi-squared test for categorical data (self-rated oral health compared with others, xerostomia, dental condition, periodontitis, and utilization and functionality of RPD and/or CD), estimating the odds ratio when appropriate. Student’s t test for independent groups were also performed.

A binary logistic regression model was used, employing frailty status as the dependent variable and the dental variables (perception of oral health compared with others, utilization of dental services during the previous year, xerostomia, report of chewing problems, number of teeth, and severe periodontitis) as independent variables. We also included interactions between history of smoking and severe periodontitis, diabetes and hospitalization, diabetes and hypertension, diabetes and severe periodontitis, diabetes and number of teeth present, myocardial infarction and hypertension, myocardial infarction and hospitalization, and hypertension and hospitalization. Utilization and functionality of RPD and CD were excluded because of their collinearity with the number of teeth. Socio-demographic and medical covariables identified as statistically significant in the univariate analysis were also included. The final model was determined by performing a backward variable selection procedure.

A p-value of 0.05 was used as the threshold for statistical significance, and 95% confidence intervals (95% CI) were estimated when appropriate. SPSS software for Windows (SPSS Inc., Chicago, IL, version 19) was used to perform all statistical tests.

## Results

The overall mean age was 77.9 ± 6.3 years; 53.2% (n = 372) were women; 67.6% (n = 221) of men were married, and 51.3% (n = 191) of women were widowed; men had completed more years of schooling (mean = 7.9 ± 5.8) than women (mean = 6.7 ± 5.0; p < 0.01).

The prevalence of frailty was 15.0% (n = 105), with 12.2% (n = 40) prevalence among men and 17.5% (n = 65) prevalence among women.

The mean number of teeth present was 10.7 ± 9.2, with a 23.5% (n = 197) prevalence of edentulism; 9.1% of the participants (n = 76) had ≥25 teeth. The utilization of RPD and/or CD among edentate or partially dentate subjects was 61.9% (n = 472); of these, 67.6% (n = 319) of RPDs and CDs were not functional. The prevalence of severe periodontitis was 8.9% (n = 57) among subjects who were partially or completely dentate (Table 1).

**Table 1:** Prevalence of oral health conditions among elderly ≥70 years old from Mexico City, 2009

**Oral health Conditions**		**n (%)**
**Self-perception of oral health compared with others of the same age**	Better	314 (37.5)
	Equal	289 (34.5)
	Worse	82 (9.8)
	Don’t Know,	153 (18.2)
	Not Answered	
	Total	838
**Utilization of dental services during the last year**	Yes	387 (46.2)
	No	450 (53.7)
	Not answered	1 (0.1)
	Total	837
**Chewing problems**	No	444 (53.0)
	Yes	393 (46.9)
	Not answered	1 (0.1)
	Total	838
**Xerostomia**	No	458 (54.7)
	Yes	378 (45.1)
	Not answered	2 (0.2)
	Total	838
**Number of teeth**	Mean (SD)	10.7 (9.2)
	Median	10
	Maximum	32
	Total	838
**Dental condition**	Edentulism	197 (23.5)
	Partial edentulism	565 (67.4)
	Dentate ≥25 teeth	76 (9.1)
	Total	838
**Utilization of RPD and/or CD among edentate and partially dentate**	Not wearing	290 (38.1)
	Wearing RPD and/or CD	472 (61.9)
	Total	762
**Functionality of RPD and/or CD among RPD and CD users**	Non functional	319 (67.6)
	Functional	153 (32.4)
	Total	472
**Periodontitis among partially and complete dentate**	Not severe	580 (91.1)
	Severe	57 (8.9)
	Rejected evaluation	4
	Total	641

RPD = Removable Partial Denture; CD = Complete Denture.

Among all subjects, 23.8% suffered from cognitive impairment; 19% suffered from urinary incontinence; 8.4% had suffered ≥3 falls in the previous year; 49.8% reported former or current smoking; 11.1% were hospitalized during the previous year; 8.4% had suffered myocardial infarction; 3.9% had suffered a stroke; 55.3% had hypertension; 21.5% were diabetics; 13.6% reported having osteoporosis; 15.6% had arthritis; and the mean number of drugs taken daily was 2.75 ± 2.2.

No significant differences were found in the prevalence of frailty based on number of falls in the previous year (p = 0.11), smoking status (p = 0.35), hypertension (p = 0.10), diabetes (p = 0.80), severe periodontitis (p = 0.29), utilization of removable or complete dentures (p = 0.19), or functionality of partial or complete dentures (p = 0.37) (Table 2).

**Table 2:** Sociodemographic, medical and oral health characteristics by frailty status, elderly ≥70 years from Mexico City, 2009

**Variable**	**Scale**	**Frailty (%)**			**p**	**OR**	**95% CI**
		**No**	**Yes**	**Total**			
**Age**	Mean (SD)	77.2 (5.9)	81.6 (6.6)	77.9 (6.3)	<0.001		
	Total	594	105	699			
**Sex**	Male	287 (87.8)	40 (12.2)	327	0.053	1.52	0.99-2.33
	Female	307 (82.5)	65 (17.5)	372			
	Total	594 (85.0)	105 (15.0)	699			
**MMSE**	19-30	523 (87)	78 (13.0)	601	<0.001	2.55	1.54-4.22
	≤18	71 (72.4)	27 (27.6)	98			
	Total	594 (85.0)	105 (15.0)	699			
**Urinary incontinence**	No	507 (88.9)	63 (11.1)	570	<0.001	3.49	2.18-5.59
	Yes	83 (69.7)	36 (30.3)	119			
	Total	590	99	689			
**≥3 falls during the previous 12 months**	No	502 (85.8)	83 (14.2)	585	0.11	1.71	0.88-3.30
	Yes	46 (78.0)	13 (22.0)	59			
	Total	548	96	644			
**Smoking**	No	282 (83.7)	55 (16.3)	337	0.35	0.82	0.52-1.25
	Yes	312 (86.2)	50 (13.8)	362			
	Total	594	105	699			
**Hospitalization previous 12 months**	No	543 (87.0)	81 (13.0)	624	<0.001	3.15	1.84-5.41
	Yes	51 (68.0)	24 (32.0)	75			
	Total	594	105	699			
**Myocardial infarction**	No	549 (85.9)	90 (14.1)	639	0.02	2.08	1.11-3.89
	Yes	44 (74.6)	15 (25.4)	59			
	Total	593	105	698			
**Stroke**	No	580 (85.7)	97 (14.3)	677	0.003	3.68	1.49-9.11
	Yes	13 (61.9)	8 (38.1)	21			
	Total	593	105	698			
**Hypertension**	No	254 (84.9)	45 (15.1)	299	1.00	1.00	0.66-1.52
	Yes	333 (84.9)	59 (15.1)	392			
	Total	587	104	691			
**Diabetes**	No	466 (84.9)	83 (15.1)	549	0.80	.936	0.56-1.57
	Yes	126 (85.7)	21 (14.3)	147			
	Total	592	104	696			
**Osteoporosis**	No	518 (87.2)	76 (12.8)	594	<0.001	2.57	1.54-4.28
	Yes	69 (72.6)	26 (27.4)	95			
	Total	587	102	689			
**Arthritis**	No	510 (86.6)	79 (13.4)	589	0.005	2.02	1.23-3.34
	Yes	83 (76.1)	26 (23.9)	109			
	Total	593	105	698			
**Utilization of dental services previous 12 months**	Yes	302 (88.3)	40 (11.7)	342	0.020	1.65	1.08-2.54
	No	292 (82.0)	64 (18.0)	356			
	Total	594	104	698			
**Xerostomia**	No	342 (87.5)	49 (12.5)	391	0.038	1.55	1.02-2.35
	Yes	252 (81.8)	56 (18.2)	308			
	Total	594	105	699			
**Chewing problems**	No	343 (88.9)	43 (11.1)	386	0.001	1.97	1.29-3.00
	Yes	251 (80.2)	62 (19.8)	313			
	Total	594	105	699			
**Severe periodontitis**	No	443 (87.7)	62 (12.3)	505	0.29	1.54	0.69-3.47
	Yes	37 (82.2)	8 (17.8)	45			
	Total	480	70	550			
**Utilization of RPD or CD**	No	185 (81.9)	41 (18.1)	226	0.19	0.75	0.48-1.16
	Yes	349 (85.7)	58 (14.3)	407			
	Total	534	99	633			
**Functionality of RPD or CD**	Yes	117 (88.0)	16 (12.0)	133	0.37	1.32	0.71-2.45
	No	232 (84.7)	42 (15.3)	274			
	Total	349	58	407			
**Number of medications used daily**	Mean (SD)	2.6 (1.9)	4.1 (2.8)	2.8 (2.2)	<0.001		
	Total	585	104	689			
**Number of teeth present**	Mean (SD)	11.8 (9.2)	8.3 (8.6)	11.3 (9.2)	<0.001		
	Median	11	6	10			
	Total	594	105	699			
**Self-perception of oral health compared with others**	Better	275 (89.0)	34 (11.0)	309	0.006		
	Same	237 (83.5)	47 (16.5)	284			
	Worse	57 (75.0)	19 (25.0)	76			
	Total	569	100	669			
**Dental condition**	Complete Edentulism	114 (76.5)	35 (23.5)	149	0.003		
	Partial Edentulism	420 (86.8)	64 (13.2)	484			
	Dentate	60 (90.9)	6 (9.1)	66			
	Total	594	105	699			

MMSE = Minimental State Examination; RPD = Removable Partial Denture; CD = Complete Denture.

The analysis also showed that frail participants were older (mean = 81.6 ± 6.6, p < 0.001), and that participants were more likely to be frail if they scored ≤18 on the MMSE (OR = 2.6; 95% CI 1.5–4.2), reported urinary incontinence (OR = 3.5; 95% CI 2.2–5.6), were hospitalized during the last year (OR = 3.2; 95% CI 1.8–5.4), had a history of myocardial infarction (OR = 2.1; 95% CI 1.1–3.9), had suffered a stroke (OR = 3.7; 95% CI 1.5–9.1), suffered from osteoporosis (OR = 2.6; 95% CI 1.5–4.3), suffered from arthritis (OR = 2.0; 95% CI 1.2–3.3), did not use dental services the previous year (OR = 1.7; 95% CI 1.1–2.5), reported xerostomia (OR = 1.6; 95% CI 1.0–2.4), or reported chewing problems (OR = 2.0; 95% CI 1.3–3.0). Further, frail subjects used a higher number of drugs on a daily basis (mean = 4.1 ± 2.8, p < 0.001) and had a lower mean number of teeth (mean = 8.3 ± 8.6, p < 0.001) than non-frail subjects. The prevalence of frailty was higher among those who perceived that their oral health was worse than others of the same age (25%) (p = 0.006) and among edentulous subjects (23.5%) (p = 0.003). (Table 2)

### Logistic regression model

The bivariate logistic regression model included gender, age, cognitive performance (MMSE), incontinence, hospitalization during the last year, history of myocardial infarction, stroke, osteoporosis, arthritis, number of drugs taken daily, self-perception of oral health, utilization of dental services during the last year, xerostomia, self-report of chewing problems, number of teeth present, and severe periodontitis.

The final model adequately describes the data (Hosmer-Lemeshow goodness-of-fit = 0.101), explains 34.4% (Nagelkerke pseudo r-squared) of the variability in frailty classification, and properly classifies 88.2% of the participants.

The final model showed an increased probability of frailty among women (OR = 1.9; 95% CI 1.07–3.5), those who reported urinary incontinence (OR = 2.7; 95% CI 1.5–4.9), those who had suffered myocardial infarction (OR = 3.8; 95% CI 1.004–14.1), those who reported their oral health to be worse than others of the same age (OR = 3.2; 95%CI 1.4–7.2), and those who did not use dental services during the previous year (OR = 2.1; 95%CI 1.2–3.7); also, for each additional year of age and each additional drug consumed per day, the probability of being frail increased 10% and 30%, respectively. Frailty prevalence was not significantly different based on hospitalization during the previous year (OR = 2.4; 95% CI 0.99–5.7, p = 0.053) or based on severe periodontitis (OR = 3.8; 95% CI 0.93–15.4, p = 0.062) (Table 3).

**Table 3:** Final logistic regression model for frailty, elderly ≥70 years old from Mexico City, 2009

	**Wald**	**DF**	**P**	**OR**	**95% CI**	
					Lower	Upper
**Sex (Women)**	4.72	1	0.03	1.94	1.07	3.52
**Age (years)**	17.62	1	0.00	1.09	1.05	1.14
**MMSE (≤18)**	2.50	1	0.11	1.72	0.88	3.39
**Incontinence (yes)**	10.66	1	0.001	2.72	1.49	4.95
**Hospitalization (yes)**	3.75	1	0.053	2.37	0.99	5.67
**Medications per day (number)**	20.09	1	0.00	1.31	1.16	1.47
**Myocardial infarction(yes)**	3.86	1	0.049	3.76	1.004	14.07
**Stroke (yes)**	1.29	1	0.26	2.14	0.57	7.98
**Osteoporosis (yes)**	2.55	1	0.11	1.76	0.88	3.51
**Self-perception of oral health**						
**Better**	8.63	2	0.01			
**Same**	3.36	1	0.07	1.76	0.96	3.24
**Worse**	8.23	1	0.004	3.23	1.45	7.21
**Utilization of dental services (no)**	6.60	1	0.01	2.10	1.19	3.71
**Number of teeth (number)**	0.65	1	0.42	0.99	0.95	1.02
**Severe periodontitis (yes)**	3.48	1	0.062	3.79	0.93	15.37
Periodontitis * smoking	0.63	1	0.43	0.43	0.05	3.51
Number of teeth * diabetes	3.01	1	0.08	0.94	0.87	1.01
Stroke * hypertension	0.63	1	0.43	0.52	0.10	2.59
Stroke * hospitalization	1.16	1	0.28	0.32	0.04	2.57
Diabetes * hospitalization	0.47	1	0.49	1.76	0.35	8.78
**Constant**	34.18	1	0.000	0.00		

MMSE = Minimental State Examination.

## Discussion

We observed, using a representative sample of people ≥70 years old who resided in one district of Mexico City, that the perception of having worse oral health than others of the same age and the failure to utilize dental services were associated with an increased probability of being frail. This is the first study that has explored frailty in the Mexican elderly and included oral health components. The multidisciplinary approach of this project allows us to explore the relationships between frailty and many other variables [32].

There are few previous data on the association between oral health and some components of the frailty syndrome, but not with the frailty syndrome overall [34,35].

There was an association between not using dental services during the past year and a higher probability of being frail and (OR = 2.1, 95% CI 1.2–3.8); Overall, the utilization of dental services among the elderly is lower [43] (49% in this study) than the utilization of medical services (85%) [44]. According to Kiyak [45], elderly people who believe that declining health is a part of aging and that recovery is not possible, reflect that attitude by not utilizing dental services. Taking this into account, the observed lack of utilization of dental services might also reflect compromised general health, which in turn may be correlated with frailty.

Another possible explanation could be constriction of life space, which has been recognized as a risk factor for frailty [46]. Constriction of life space is attributable to loss of mobility, weakness, and slowness, which result in difficulties with moving and travelling independently to receive dental care. This, in addition to low expectations about oral health, could contribute to this population’s failure to utilize dental services.

The probability of being frail was 2.2 times higher for those who considered their oral health worse than others of the same age; this could be explained by the positive association between oral health and general health [3,6]. We can assume that a bad perception of oral health reflects declining general health.

Oral health is recognized as a component of general health [3], and it is unusual for people to identify oral health conditions as their only health problems when those issues are so advanced that they affect feeding ability, speaking, chewing, physical appearance, and social life, frequently producing pain and favoring depression [47-50]. Also, when oral health is poor, inflammation markers are increased, which in turn can alter the metabolism of other organs; even though self-perception of oral health is subjective, it can represent a risk indicator for frailty.

Oral health expectations among the elderly could also be influenced by age, education level, socioeconomic situation, and social support [51], variables that have been associated with frailty.

Even though the presence of severe periodontitis did not surpass the threshold of statistical significance as a predictor of frailty (OR = 3.9, 95% CI 0.98–15.6) in the final logistic model, the physiological process related to the development of periodontal infection could be related to the development of frailty. It has been shown that severe periodontitis is linked to energy imbalance, which in the elderly has been associated with loss of mobility and strength alterations that end with the development of frailty syndrome. It has been shown that higher concentrations of pro-inflammatory cytokines could favor the altered inflammation state observed in frailty syndrome [29,52,53]. It is important to point out that periodontitis requires the presence of teeth; in the logistic regression model, we included edentulous subjects in the “no severe periodontitis” group in order to preserve the sample size, but in a later analysis considering only completely or partially dentate (1–32 teeth) subjects, we observed that those with severe periodontitis have 5.3 times the risk for frailty than those without severe periodontitis (95% CI 1.3–22.2). The participation of severe periodontitis in the development of frailty should be explored in the longitudinal analysis.

Other studies have explored the relationship between dental variables and frailty: Avlund et al. [34] reported that having few teeth increases the risk of fatigue in the elderly; also, Semba et al. [35] reported that frail edentate women with complete dentures who complain about chewing problems have higher mortality rates than frail edentulous women who reported no chewing problems. In this study, we did not find an association between the number of teeth and frailty or between chewing problems and frailty; we must consider that in our sample the prevalence of overweight and obesity was 75%, which could dissipate the effect of tooth loss on frailty among the elderly in Coyoacán. On the other hand, elderly people living in Mexico who have few (1-9) teeth and who wear nonfunctional dental prostheses tend to modify their diets, increasing the consumption of low-nutrient foods. This could explain the fact that many of them are overweight and obese, and it could hide the effects of having few or no teeth on the risk of frailty and mortality [34,35]. It is important to mention that obese sarcopenia could be a confounding factor in the evaluation of frailty [54].

Several researchers have measured dental variables with physical activity. Takata and cols., explored the association between chewing ability and number of teeth with physical activity using hand grip strength, leg extensor strength, leg extensor power, stepping rate, and one-leg standing time as indicators. These are specific measurements of physical activity, one of the components of frailty. However, comparisons with our study are difficult because we used The Physical Activity Scale for the Elderly (PASE) [38], an instrument designed for screening for low physical activity in the elderly. Also, we measured frailty as a whole and not considering the components individually. Even when the associations of chewing ability and number of teeth with frailty were statistically significant in the univariate model, in the logistic regression model the associations disappeared. Similarly, Takata did not find association with number of teeth; but when comparing the number of chewable foods they observed an increase in isokinetic leg extension power and in one-leg standing time, association that we did not observed with frailty. The type of measurement for physical activity used in this study might be a limitation, it is worth considering in future studies a more detailed measurement [55].

It is important to mention that this study included clinical evaluation of dental variables as well as questions about self-perception of oral health and self-reports of utilization of dental services during the last year. None of these variables has been considered in other reports on frailty. However, the inclusion of several oral health conditions represents a challenge because of the mutually exclusive characteristics of some dental variables (e.g., periodontitis and edentulism).

We recognize the need for a global oral health indicator, and we question, “What would be an optimal oral health indicator in the elderly, and how can we measure it?” A global oral health indicator should be able to classify those people who are properly rehabilitated (using functional dental prosthesis) and have no periodontal problems. It should also be able to classify those with complex oral conditions (e.g., nonfunctional dental prosthesis and periodontal disease in the remaining teeth, or presence of remaining roots), which could increase the risk of developing other chronic conditions, favoring energy imbalance and creating clinical and therapeutic challenges.

The covariates measured in this analysis were consistent with those measured in previous reports; we observed a higher risk of frailty among those who had been hospitalized during the previous year, those who consumed higher number of drugs on a daily basis, those who reported incontinence, and those who had a history of myocardial infarction [26,27,51,56-58].

### Study limitations

Due to the cross-sectional nature of the study design, causal inferences cannot be made. In order to deepening in the relationship between oral health status and frailty, it is necessary to perform longitudinal studies.

Even though the non-response rate was 25% for the dental clinical evaluation, many characteristics of those who accepted and those who did not accept the oral clinical evaluation were similar. Similarities between responding and non-responding groups were found with respect to sociodemographic characteristics such as age and gender, medical variables, dental services utilization, and self-perception of oral health. The number of years of education completed was higher among those who did not accept the oral clinical evaluation. As a whole, these results imply that there are no differences between subjects who were clinically evaluated and those who were not. Certain oral health-related variables, such as coronal and root caries and oral mucosal lesions, were not evaluated because of time constraints on the clinical evaluations. Furthermore, it is possible that the mutually exclusive relationships between some clinical dental data (e.g., periodontitis and edentulism) interfere with the inclusion of the variables in the analysis. Another limitation could be the type of measurements used for frailty; we used epidemiological screening methods recommended for the elderly population. In order to have more accurate results it can be considered the utilization of more detailed measurements for the components of frailty in future studies.

## Conclusions

Low utilization of dental services and poor self-perception of oral health could be considered possible risk markers for frailty syndrome. The role of periodontitis and other oral clinical variables in frailty should be explored in longitudinal studies. The comprehensive geriatric assessment should include a question about self-perception of oral health, because this question reflects both objective and subjective aspects of health and is associated with a higher probability of being frail. The inclusion of a question about utilization of dental services during the previous year on the comprehensive geriatric assessment should also be considered, because the answer to this question is related to other factors that can promote the development of frailty.

## Abbreviations

CD: Complete Denture; INCMNSZ: Instituto Nacional de Ciencias Médicas y Nutrición “Salvador Zubirán” (Institute of Medical Sciences and Nutrition “Salvador Zubirán”); INSP: Instituto Nacional de Salud Pública (National Institute of Public Health); MMSE: Mini-Mental State Examination; PSR: Periodontal Screening and Recording Index; RPD: Removable Partial Denture; SD: Standard Deviation; UNAM: Universidad Nacional Autónoma de México (National Autonomous University of Mexico); WHO: World Health Organization.

## Competing interests

The authors declare that they have no competing interests.

## Authors’ contributions

SABY, LMGR, and JAAF participated in the design of the study. RCCP and SABY performed data analysis, interpreted the data and drafted the manuscript. All authors read and approved the final manuscript.

## Pre-publication history

The pre-publication history for this paper can be accessed here:

http://www.biomedcentral.com/1471-2458/12/773/prepub
